# Intermittent Versus Continuous Low-Energy Diet in Patients With Type 2 Diabetes: Protocol for a Pilot Randomized Controlled Trial

**DOI:** 10.2196/21116

**Published:** 2021-03-19

**Authors:** Sarah McDiarmid, Michelle Harvie, Rhona Johnson, Avni Vyas, Azza Aglan, Jacqui Moran, Helen Ruane, Amanda Hulme, Katharine Sellers, Basil Issa

**Affiliations:** 1 The Prevent Breast Cancer Research Unit Manchester University NHS Foundation Trust Manchester United Kingdom; 2 Department of Endocrinology and Diabetes Manchester University NHS Foundation Trust Manchester United Kingdom; 3 Greater Manchester Mental Health Foundation Trust Manchester United Kingdom

**Keywords:** type 2 diabetes, diabetes, diabetic diet, low-energy diet, low calorie diet, intermittent energy restriction, intermittent fasting, diabetes remission, smartphone, mobile phone, mHealth, mobile health

## Abstract

**Background:**

Intensive face-to-face weight loss programs using continuous low-energy diets (CLEDs) providing approximately 800 kcal per day (3347 kJ per day) can produce significant weight loss and remission from type 2 diabetes (T2D). Intermittent low-energy diets (ILEDs) and remotely delivered programs could be viable alternatives that may support patient choice and adherence.

**Objective:**

This paper describes the protocol of a pilot randomized controlled trial to test the feasibility and potential efficacy of remotely supported isocaloric ILED and CLED programs among patients with overweight and obesity and T2D.

**Methods:**

A total of 79 participants were recruited from primary care, two National Health Service hospital trusts, and a voluntary T2D research register in the United Kingdom. The participants were randomized to a remotely delivered ILED (n=39) or CLED (n=40). The active weight loss phase of CLED involved 8 weeks of Optifast 820 kcal/3430 kJ formula diet, followed by 4 weeks of food reintroduction. The active weight loss phase of ILED (n=39) comprised 2 days of Optifast 820 kcal/3430 kJ diet and 5 days of a portion-controlled Mediterranean diet for 28 weeks. Both groups were asked to complete 56 Optifast 820 kcal/3430 kJ days during their active weight loss phase with an equivalent energy deficit. The diets were isocaloric for the remainder of the 12 months. CLED participants were asked to follow a portion-controlled Mediterranean diet 7 days per week. ILED followed 1-2 days per week of a food-based 820 kcal/3430 kJ diet and a portion-controlled Mediterranean diet for 5-6 days per week. Participants received high-frequency (weekly, fortnightly, or monthly depending on the stage of the trial) multidisciplinary remote support from a dietitian, nurse, exercise specialist, and psychologist via telephone or the Oviva smartphone app. The primary outcomes of the study were uptake, weight loss, and changes in glycated hemoglobin at 12 months. An outcome assessment of trial retention was retrospectively added. Secondary outcomes included an assessment of adherence and adverse events. A qualitative evaluation was undertaken via interviews with participants and health care professionals who delivered the intervention.

**Results:**

A total of 79 overweight or obese participants aged 18-75 years and diagnosed with T2D in the last 8 years were recruited to the Manchester Intermittent and Daily Diet Diabetes App Study (MIDDAS). Recruitment began in February 2018, and data collection was completed in February 2020. Data analysis began in June 2020, and the first results are expected to be submitted for publication in 2021.

**Conclusions:**

The outcomes of the MIDDAS study will inform the feasibility of remotely delivered ILED and CLED programs in clinical practice and the requirement for a larger-scale randomized controlled trial.

**Trial Registration:**

International Standard Randomized Controlled Trial Number (ISRCTN) 15394285; http://www.isrctn.com/ISRCTN15394285

**International Registered Report Identifier (IRRID):**

DERR1-10.2196/21116

## Introduction

### Background

An estimated 4.7 million people have type 2 diabetes (T2D) in the United Kingdom, with the number expected to rise to over 5.5 million by 2030 [1]. Diabetes related complications are common, and people with T2D die up to 10 years earlier than those without the disease [2]. Currently, 10% of the National Health Service (NHS) budget in the United Kingdom is spent on diabetes (approximately £10 billion (US $13.8 billion) per year) [1].

Approximately 80% to 90% of people with T2D have overweight or obesity [3-6]. Clinical guidelines for the management of T2D focus largely on multiple drug treatments to reduce blood glucose. They also recommend at least 5% to 10% weight loss [7,8] as this leads to improvements in glycemic control, insulin sensitivity, blood lipids, and blood pressure (BP) [9,10].

### Continuous and Intermittent Low-Energy Diets

Intensive face-to-face weight loss programs using continuous low-energy diets (CLEDs) that provide approximately 800 kcal (3347 kJ) per day of formula-based total diet replacement for 8 to 20 weeks or longer are highly effective for large weight loss and remission from T2D [11-14]. The recently published Diabetes Remission Clinical Trial (DiRECT) tested an intensive CLED program in primary care and found that it was superior to standard best practice care (standard daily moderate energy restriction advice with minimal support). At 12 months, 45.6% (68/149) participants of the intervention group achieved remission with an average weight loss of 10 kg compared with 4.0% (6/149) and 1 kg loss in the control group (*P*<.001). Remission was highest (31/36, 86%) in those who achieved 15% weight loss [13] and is more likely in those diagnosed with T2D in more recent years [15].

The CLED approach using total diet replacement is thought to be effective because the initial rapid weight loss can be highly motivating [16], and formula diets remove decision making around food choices. In addition, subjective hunger may be reduced by the associated ketosis [17]. CLEDs have been shown to reduce excess fat in the liver and pancreas in patients with T2D, which is part of the proposed mechanism for T2D remission [11,18].

Possible drawbacks of the CLED approach are that it is not appealing to or achievable for everyone. Participants following CLED programs report shame and awkwardness in social situations centered around food [19]. Attrition in intensive CLED studies on people with overweight or obesity (+/−T2D) is approximately 25% [13,20], although higher rates have been reported in studies with less frequent health care professional (HCP) contact [14]. The prevention of weight gain following a CLED program remains a key challenge. In 2 years, participants in the CLED group in the DiRECT trial appeared to have regained approximately 40% of the weight they had lost after the initial total diet replacement phase despite regular face-to-face support and an intensive 2-year relapse program involving repeated spells of a CLED, partial meal replacements, and antiobesity medications [13,21].

An intermittent low-energy diet (ILED) is a potential alternative to CLED. This includes the same number of low-energy formula diet days as CLED, but these days are undertaken for 2 days per week over a longer period. ILED may provide an alternative approach for people who find CLEDs unappealing or difficult to maintain. Qualitative reports on people following CLEDs suggest that they would prefer an intermittent approach that may be easier to fit into life without the need for weeks away from normal food [22].

A recent randomized controlled trial (RCT) in people with T2D compared an ILED with regular, daily, modest energy restriction (1200-1500 kcal/5020-6276 kJ per day) for 12 months and showed similar improvements to glycemic control in both groups [23]. Early studies of CLEDs show that some of the improvements in insulin sensitivity and beta cell function are associated with acute energy restriction rather than weight loss [24]. As these benefits subsequently attenuate when subjects enter weight maintenance with euenergetic feeding [25], ongoing spells of intermittent energy restriction each week may be a way to maintain beneficial glycemic control. A recent RCT (n=46) of participants with prediabetes and overweight or obesity showed that an ILED every other day for 12 months produced greater reductions (*P*<.05) in fasting insulin −52% (SE 9%) and insulin resistance -53% (SE 9%) compared with isocaloric daily moderate calorie restriction (−14%, SE 9%; −17%, SE 11%) despite similar decreases in body weight [26].

An ILED with 2 low-energy days per week will lead to a slower initial weight loss compared with a CLED. An unanswered research question is whether an ILED may lead to improved weight loss maintenance compared with daily dietary approaches in patients with T2D.

The relative benefits of ILED versus isocaloric CLED regarding glycemic control, diabetes remission, and weight loss maintenance in people with T2D are unknown.

### Remote Follow-up

A potential strategy for increasing adherence efficacy and reach of low-energy diet (LED) programs may be to include high-frequency remote follow-up, which has been shown to be superior to low frequency face-to-face care in weight management interventions [27]. Remote care reduces participants’ burden of attending face-to-face appointments and may be cost-effective compared with face-to-face care [28] while improving access to care. There is growing evidence to support the use of telehealth (including telephone and mobile phone–based apps) to monitor and provide feedback to patients with T2D and promote self-management of their condition [29-31].

The Manchester Intermittent versus Daily Diet Diabetes App Study (MIDDAS) incorporated high-frequency remote follow-up via the Oviva smartphone app and by telephone [32]. The app was used to facilitate self-monitoring of diet, weight, and blood glucose, and communication with HCPs, with the option of remote peer group support. Group participation in mobile apps has been shown to predict weight loss success [33]. Remote communities can be encouraging, motivating, and informative while remaining convenient and anonymous [34].

### Goals of This Study

The primary aim of MIDDAS is to assess the feasibility and potential efficacy of remotely supported ILED and isocaloric CLED programs in patients with overweight or obesity and T2D. The feasibility of an RCT comparing the 2 approaches was also assessed. MIDDAS did not have a control group for comparison because CLED programs have already been shown to be superior to standard or best practice.[13] The initial estimates of acceptability (uptake and retention) and potential efficacy (change in weight and glycated hemoglobin [HbA_1c_]) of the programs will determine whether progression to a full RCT is indicated and will inform the feasibility of delivering ILED and CLED programs that incorporate remote follow-up.

## Methods

The trial protocol (V5.0/08.04.19) was granted ethical approval by the North West Greater Manchester South Research Ethics Committee (ref:17/NW/0389). SPIRIT reporting guidelines were used [35].

### Design and Setting

The study was a 12-month pilot 2-arm RCT, performed in patients with T2D and overweight or obesity, recruited from general practices, NHS hospital trusts and an NHS-supported voluntary research register in England. Participants attended trial assessments at Manchester University NHS Foundation Trust (MFT), Manchester, United Kingdom. Participants received dietary support remotely via the Oviva app and/or by telephone.

### Recruitment

Potential eligible participants were recruited from 3 settings using the following methods:

1. Patients in three general practices in Manchester were sent targeted invite letters and a text message reminder 8 weeks later if there was no response. These practices included a population of patients with T2D ranging from 400 to 700 and reflected different levels of deprivation in England. The England Index of Multiple Deprivation (IMD) score for each practice was 10.9 (least deprived 25% in England) and 29.8 and 44.1 (top 25% deprived in England) [36].
2. Patients at MFT and Stockport NHS Foundation Trust were invited to the study via patient record search and invitation letter, using poster displays, or during face-to-face routine clinical contacts.
3. Patients with T2D (n=2500) on the Help BEAT Diabetes volunteer database (hosted by the National Institute of Health Research Clinical Research Network for Greater Manchester) were contacted via mail or email and asked to check if they met the eligibility criteria for the trial and to contact the MIDDAS trial team if they were interested in taking part.

Those invited by letter had the opportunity to tell the trial team why they did not wish to take part via an anonymous reply slip. Interested patients were invited to an optional group session at MFT to receive more information on the diets and try the Optifast meal replacements before deciding to participate.

The inclusion and exclusion criteria for the trial is detailed in Textbox 1.

**Inclusion and exclusion criteria.**

Inclusion criteria

- Willing and able to provide written informed consent
- Male or female aged between 18 and 75 years
- Diagnosed with type 2 diabetes <8 years
- Diet controlled only or receiving any type of diabetes medications including insulin
- Glycated hemoglobin (HbA_1c_) ≥48 mmol per mol (6.5%) at baseline (venous blood sample)
- BMI>27 kg per m^2^ and <50 kg per m^2^ or >25 kg per m^2^ and <50 kg per m^2^ in high-risk ethnic minority groups (ie, South Asian, Black African, and African Caribbean)
- Access to and ability to use the telephone
- Willing to be randomized to an intermittent low-energy diet or continuous low-energy diet using total diet replacement drinks

Exclusion criteria

- Routine HbA_1c_ ≥108 mmol per mol (12.0%) during the last 3 months
- Unstable retinopathy, grade R2 or higher, or no retinopathy screen within the last 12 months
- Pregnant or considering pregnancy
- Previous bariatric surgery
- Current treatment with Orlistat
- Unintentional weight loss ≥5 kg within last 6 months
- Learning difficulties, lacking capacity or unable to understand English
- Known sensitivity to ingredients in the total diet replacement
- Diagnosed eating disorder. Severe binge eating or very low eating self-efficacy were assessed using the following questionnaires: Binge Eating Scale (BES [37], score ≥27) and Weight Efficacy Lifestyle Questionnaire Short Form (WEL-SF [38], score ≤35)
- Severe anxiety or depression was assessed using the Generalized Anxiety Disorder Scale (GAD-7 [39], score ≥15) and Patient Health Questionnaire-9 (PHQ-9[40], score ≥15). Hazardous or harmful drinking was indicated by the Alcohol Use Disorders Identification Test (AUDIT [41], score ≥16)
- Active symptoms associated with emotionally unstable personality disorder, bipolar disorders, psychotic disorders, post-traumatic stress disorder, and current self-harm or suicidal behavior. Participants with these issues were potentially eligible, dependent on further information from their general practitioners and responses to the baseline study questionnaires
- Current treatment with lithium, antipsychotics, or other psychotropic medications that may cause excessive weight gain
- Chronic use of steroids
- Medical conditions that in the opinion of the treating physician were at risk of deterioration (eg, severe systemic or organ disease, active cancer, liver, gall bladder disease, and pancreatitis)
- Current participation in a diabetes drug trial

### Participant Flow and Medication Management

**Figure 1:**
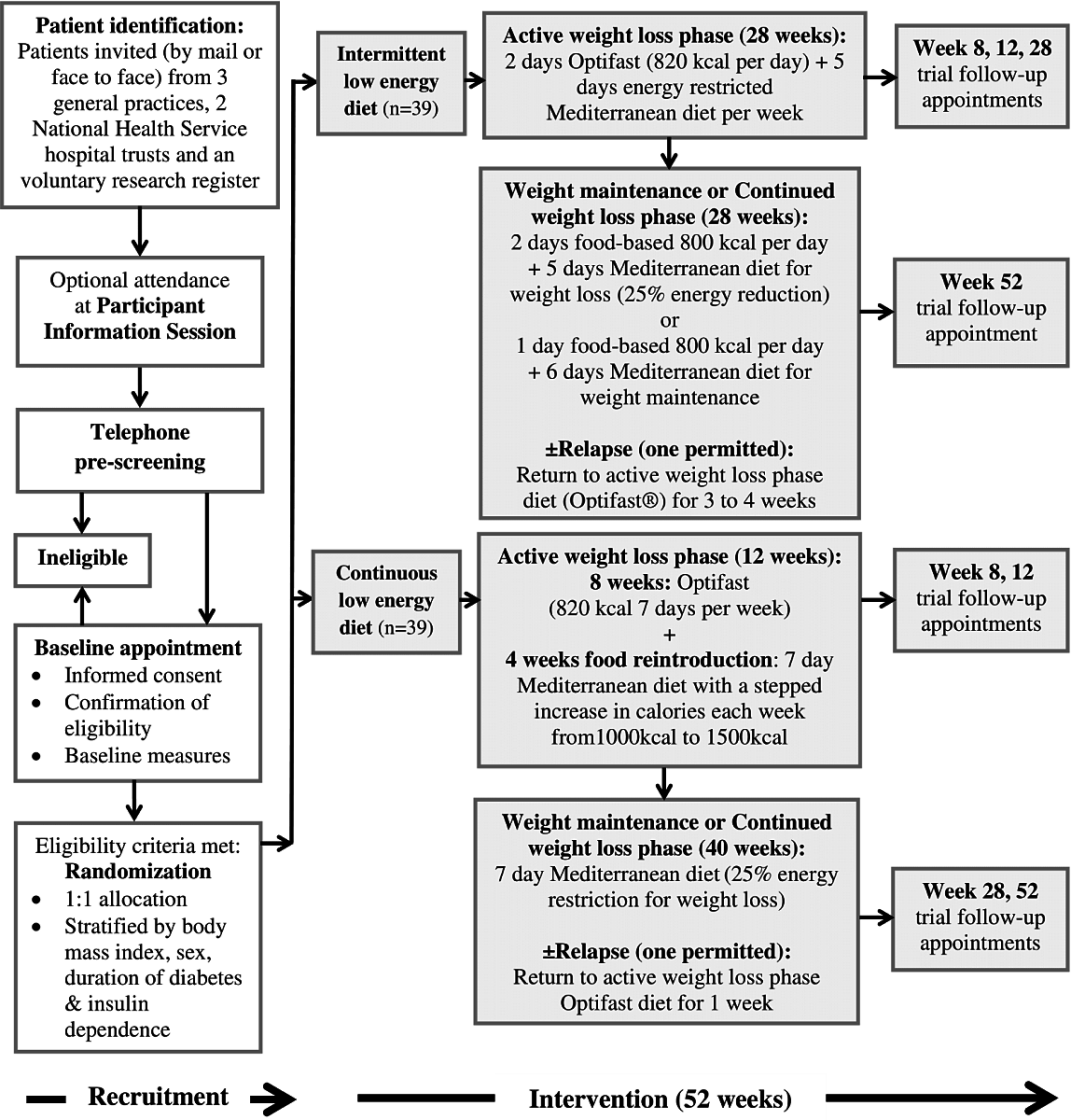
Participant flow through study.

The participant flow through the study is outlined in Figure 1. Informed consent was obtained by the trial research nurse at the baseline appointment. Eligible participants were randomized to ILED or CLED. All participants were invited to attend follow-up appointments at MFT for a repeat of clinical measurements at weeks 8, 12, 28, and 52.

Changes to diabetes and antihypertensive medications specific to each treatment arm are detailed in the trial medication management plan in Table S2 of Multimedia Appendix 1. The CLED medication management protocol was devised by the research team and instructed participants to stop all diabetes medications, with the exception of metformin. Insulin was stopped or reduced, depending on the baseline HbA_1c_ level. Medication management of the ILED arm was adapted from a protocol tested in a recent ILED trial [42]. Medications in both arms were reintroduced or titrated during the trial according to clinical needs. General practitioners (GPs) were notified of the enrollment of patients and changes to their medications by letter.

### Randomization and Blinding

Eligible participants were randomized 1:1 to ILED or CLED by a researcher independent of the intervention using a minimization program stratified by BMI ≥34 or <34 kg per m^2^ (projected mean value from the counterpoint and counterbalance studies [11,12,15]), sex, duration of diabetes <4 years or ≥4 years, and whether participants were prescribed insulin. Due to the nature of the intervention, it was not possible to blind participants and clinicians to the treatment allocation. Clinical assessments were performed by an independent research assistant. Laboratory results were assessed by independent laboratory staff.

### Interventions

Figure 1 shows a summary of the 2 dietary programs. Both included a combination of the Optifast LED and the Mediterranean diet over a period of 12 months. Both programs were designed to have exactly 56 Optifast LED days and an equivalent level of energy restriction during their active weight loss phase and weight maintenance or continued weight loss phase.

#### CLED

##### Active Weight Loss Phase (12 weeks)

Weeks 1 to 8 involved the Optifast LED (Néstle Health Science, United Kingdom). This provided approximately 820 kcal (3430 kJ) per day and consisted of 3 sachets per day of Optifast (200 kcal/837 kJ per sachet of shake or soup made with water) in addition to 8×80 g portions of nonstarchy vegetables (approximately 140 kcal/586 kJ), one dessertspoon of oil per day (80 kcal per 335 kJ), and 2-2.5 liters of calorie-free fluids. The participants were asked to avoid alcohol and excessive caffeine to minimize the risk of dehydration. Participants who were unable to tolerate Optifast were offered a food-based LED with a similar macronutrient profile. This comprised 250 g of lean protein foods (eg, lean meat, fish, eggs, and vegetarian proteins), 5 portions of nonstarchy vegetables, 3 portions of low-fat dairy (eg, 200 ml milk), one portion of unsaturated fat (eg, small handful nuts), one portion of fruit, and one carbohydrate portion (eg, slice of bread). Participants reported their adherence to the LED days throughout the trial during their regular contact with the dietitian.

Diet reintroduction in weeks 9-12 allowed a food-based, energy-restricted Mediterranean diet providing 1000 kcal (4184 kJ) daily in week 1, 1200 kcal (5021 kJ) daily in week 2, 1400 kcal (5858 kJ) daily in week 3, and 1500 kcal (6276 kJ) daily in week 4.

The Mediterranean diet, as described previously by Harvie et al [43], was relatively high in protein (25% energy) with moderate carbohydrate (45% energy from low glycemic load carbohydrates), moderate fat (30% energy from fat: 15% monounsaturated, 8% polyunsaturated, and 7% saturated fatty acids), and limited alcohol to <10 units per week.

##### Weight Maintenance or Continued Weight Loss Phase (40 Weeks)

Participants who achieved a trial weight loss goal of 15%, and/or their target weight if greater, were advised to follow a euenergetic Mediterranean diet for weight maintenance. Those who had not achieved 15% weight loss or wished to lose more weight were asked to follow a 25% energy-restricted Mediterranean diet. Individuals’ estimated energy requirements were calculated using the Mifflin equations [44] to estimate the basal metabolic rate×reported metabolic equivalent of the task. These calculations were based on the revised weight and activity levels after the completion of the active weight loss phase.

#### ILED

##### Active Weight Loss Phase (28 weeks)

The ILED group was asked to include 2 consecutive days per week of the Optifast LED plus 5 days of a Mediterranean diet for 28 weeks. An energy deficit was applied to the Mediterranean diet (up to a value of 265 kcal [1109 kJ] per day) to ensure that the ILED and CLED diets were isocaloric during the active weight loss phase.

##### Weight Maintenance or Continued Weight Loss Phase (24 weeks)

Participants were asked to follow a food-based ILED. Participants who had achieved 15% weight loss, and/or their target weight if greater, were asked to follow the food-based LED described above for one day per week and a euenergetic Mediterranean diet aimed at weight maintenance (eg, 2550 kcal [10,669 kJ] per day for a 50-year-old man, BMI 34) for 6 days. Participants who had not achieved 15% weight loss or wished to lose more weight were asked to follow the food-based LED for 2 consecutive days per week and an energy-restricted Mediterranean diet (eg, 2100 kcal [8786 Kj] per day for a 50-year-old man, BMI 34) for 5 days. This provided an overall daily 25% energy restriction through the week.

#### Relapse

Trial participants who regained 2 kg or more during their weight maintenance or continued weight loss phase were offered one opportunity to resume their initial active weight loss diet to help reverse the weight gain. The CLED group was offered the 820 kcal (3430 kJ) formula Optifast LED for one week, and the ILED group was offered the Optifast LED 2 days per week for 3 to 4 weeks. The relapse program included increased support and monitoring by the trial dietitian and support from the trial psychologist to explore reasons for weight regain and to prevent further relapse.

#### Dietitian and Nurse

The diabetes specialist dietitian provided education on the diets and how to use the Oviva app, either by telephone or face-to-face. All participants were provided with written support materials, including recipes and meal plans. The diabetes specialist nurse provided support for patients who were on diabetes medications other than metformin, on antihypertensives, or who were hypertensive at baseline. All changes to diabetes and antihypertensive medications were agreed upon by the trial doctor and communicated to participants and their GP by a diabetes specialist nurse.

#### Physical Activity

Participants were encouraged to undertake physical activity (PA) throughout the trial to limit the loss of fat-free mass and promote weight loss maintenance. PA was supported by an exercise specialist, and suitability for PA was assessed at baseline using the Physical Activity Readiness Questionnaire (PAR-Q) [45] and signed off by the trial consultant endocrinologist. Participants were encouraged to aim for 5×30 minutes of moderate-intensity cardiovascular PA per week and resistance exercises for the legs, arms, and trunk 3 times per week. They were signposted to local PA services as appropriate and were educated on minimizing the risk of hypoglycemia. Diabetes medications were managed alongside exercise in response to the reported blood glucose readings.

#### Psychological Support

Enhanced psychological support from the trial psychologist was available for participants whose baseline scores indicated moderate scores for binge eating (score 18-26 on BES [37]), self-efficacy (score 36-45 on the Weight Efficacy Lifestyle Questionnaire Short Form (WEL-SF) [38]), anxiety (score 10-14 on the generalized anxiety disorder scale (GAD-7, [39]), depression (score 10-14 on the Patient Health Questionnaire-9 [PHQ-9]; [40]), or risk of alcohol-related problems (score 8-15 on the Alcohol Use Disorders Identification Test [AUDIT]; [41]). Psychological support was also available to participants who relapsed or had been identified by the team as experiencing difficulties impairing their ability to adhere to the programs. Psychological intervention was informed by motivational interviews, cognitive behavioral therapy, behavioral activation, mindfulness, emotional regulation, and distress tolerance skills.

#### Remote Behavioral Support

All participants received regular remote support from a multidisciplinary team including a diabetes specialist dietitian, nurse, and exercise specialist. The frequency and mode of behavioral support are detailed in Tables S1 and S2 in Multimedia Appendix 2. For example, the dietitian contacted the participants weekly via Oviva app messaging in weeks 1 to 12, fortnightly in weeks 13 to 28, and monthly in weeks 29 to 52. Follow-up telephone calls with the dietitian were performed at weeks 8, 12, 28, and 52.

Participants were invited to communicate with the multidisciplinary team via the Oviva app functional on iOS and Android smartphones and tablets. The app facilitates written messages and self-monitoring of diet, weight, blood glucose, weight, and activity levels. Participants were also invited to take part in group messaging on the app with other participants from their allocated diet group. Use of the app was optional, so if participants chose not to use it, then their scheduled contacts were done by telephone.

Participants signed a treatment contract to monitor their blood glucose and BP according to the protocol (Table S1 in Multimedia Appendix 1) and reported these values by telephone or via the Oviva app to the multidisciplinary team. The number of participants in both arms who requested face-to-face dietary consultations during the trial was recorded.

The multidisciplinary team was trained in motivational interview techniques (a well-established model of supporting behavioral change with proven efficacy in facilitating weight loss) to support dietary behavioral changes during both LED programs and in the longer term. Both programs used behavior change techniques such as *problem solving* and *feedback on behavior*, identified in a recent systematic review as being effective in reducing HbA_1c_ [46]. The programs also used established behavior change techniques, such as goal setting and self-monitoring [47].

### Outcomes

#### Primary

1. Uptake: To achieve an uptake of at least 10% from a primary care mail out.
2. The proportion of subjects in the ILED and CLED groups who successfully lost and maintained >15% weight loss at 12 months, as determined by intention-to-treat (ITT) analysis.
3. The proportion of subjects in both groups who achieved HbA_1c_ <48 mmol per mol (6.5%) at 12 months using ITT analysis.
4. Retention: Aiming for a retention of 60% (48/79) completion as measured by attendance at the 12-month appointment. This is the acceptable completion rate in NICE guidance for commissioning weight management services in England [48].

#### Secondary

##### Process Measures

1. Participant adherence to the protocol including self-reported adherence to LED days, preference for face-to-face contact with the dietitian, preference for food-based LED days over Optifast, and attendance at follow-up appointments.
2. Download and usage of the Oviva app for self-monitoring.

##### Exploratory Measures

Change in the following measures across the 12-month study period:

1. Body fat and fat-free mass (bioelectrical impedance)
2. Waist and hip circumference
3. BP, lipid profile, and fasting blood glucose levels
4. Number and dosage of diabetes and BP medications
5. Self-efficacy for eating, anxiety, depression, and quality of life (as measured by WEL-SF [38], GAD-7 [39], PHQ-9 [40] and Audit of Diabetes-Dependent Quality of Life (ADDQoL) [49] questionnaires, respectively)
6. Quality of diet on non-LED days (as measured by the Mediterranean diet score questionnaire [50])
7. PA (as measured by the Scottish Physical Activity Questionnaire [51])
8. Self-reported satisfaction with weight loss was measured using a 7-point Likert scale. This is highly relevant for the comparison of ILED and CLED, where the CLED will experience faster initial weight loss and achievement of their weight loss goal than the ILED arm [52]
9. Serious adverse events reported up to the end of the 12-month trial

### Measurements

Table 1 provides a summary of the measurements collected at baseline and weeks 8, 12, 28, and 52 by a research nurse.

**Table 1:** Schedule of enrollment and assessments.

Schedule		Enrollment	Follow-up visits			
		Baseline	8 weeks	12 weeks	28 weeks	52 weeks
**Enrollment**						
	Informed consent	✓^a^	—^b^	—	—	—
	Eligibility screen	✓	—	—	—	—
	Randomization	✓	—	—	—	—
**Assessments**						
	Height	✓	—	—	—	—
	Weight	✓	✓	✓	✓	✓
	Waist circumference	✓	✓	✓	✓	✓
	Hip circumference	✓	✓	✓	✓	✓
	Body fat or fat-free mass (impedance)	✓	✓	✓	✓	✓
	Blood Pressure, heart rate^c^	✓	✓	✓	✓	✓
	Blood lipids, liver function, renal profile	✓	—	—	✓	✓
	Fasting plasma glucose	✓	✓	✓	✓	✓
	Laboratory HbA_1c_^d^	✓	—	✓	✓	✓
	Pregnancy urine test	✓	✓	✓	✓	✓
	BES^e^ questionnaire	✓	—	—	✓	✓
	AUDIT^f^ questionnaire	✓	—	—	✓	✓
	WEL-SF^g^ questionnaire	✓	—	—	✓	✓
	PHQ-9^h^ questionnaire	✓	✓	✓	✓	✓
	GAD-7^i^ questionnaire	✓	✓	✓	✓	✓
	EQ-5D-3L^j^ questionnaire	✓	—	—	✓	✓
	ADDQoL^k^ questionnaire	✓	—	—	✓	✓
	Mediterranean diet score	✓	—	✓	✓	✓
	S-PAQ^l^, PAR-Q^m^	✓	—	✓	✓	✓
	Self-satisfaction with the weight loss question^n^	—	✓	✓	✓	✓
	Participant qualitative interviews	—	—	✓ (CLED^o^)	✓ (ILED^p^)	—
	Health care professional qualitative interviews	—	—	—	—	✓

^a^Event or assessment occurred at this time point.

^b^Event or assessment did not occur at this time point.

^c^Further investigation with an ECG if heart rate <50 beats per minute and not on beta-blockers.

^d^HbA_1c_: glycated hemoglobin.

^e^BES: Binge Eating Scale.

^f^AUDIT: Alcohol Use Disorders Identification Test.

^g^WEL-SF: Weight Efficacy Lifestyle Questionnaire Short Form.

^h^PHQ-9: Patient Health Questionnaire scale-9.

^i^GAD-7: Generalized Anxiety Disorder scale-7.

^j^EQ-5D-3L: Measure of health-related quality of life.

^k^ADDQoL: Audit of Diabetes-Dependent Quality of Life.

^l^S-PAQ: Scottish Physical Activity Questionnaire.

^m^PAR-Q: Physical Activity Readiness Questionnaire.

^n^Given the effort you put into following the diet and exercise plan, how satisfied are you with the amount of weight you have lost or gained during the past month? 1=very dissatisfied to 7=very satisfied.

^o^CLED: continuous low-energy diet.

^p^ILED: intermittent low-energy diet.

#### Participant Characteristics

At baseline, we collected information on participants’ age, sex, marital status, number of dependents living at home, ethnicity, education history and employment status, IMD score based on their postal code, relevant medical history, and current medications.

#### Physical Measurements

Weight and body composition were measured using Tanita BC-300MA calibrated scales to the nearest 0.1 kg. Height was measured using a portable stadiometer. Waist circumference was measured halfway between the point of the lowest rib and the iliac crest, and hip circumference was measured at the maximum circumference of the buttocks [53]. All measurements were taken to the nearest 1 mm. BP was measured with patients seated at rest for at least 10 minutes. All assessors were trained in accordance with the departmental protocols.

#### Fasting Blood Sample

A fasting venous blood sample was collected for HbA_1c_, plasma glucose, lipid profile, serum urea and electrolytes, eGFR, creatinine, and liver function tests. A urine pregnancy test was performed in women of childbearing age to exclude pregnancy. Pregnant women were excluded from the trial. Laboratory results were assessed by independent laboratory staff.

#### Questionnaires

The full list of questionnaires referenced in Table 1 is BES [37], WEL-SF [38], GAD-7 [39], PHQ-9 [40], AUDIT [41], PAR-Q [45], ADDQoL [49], Mediterranean diet score [50], Scottish Physical Activity Questionnaire [51], and EQ-5D-3L [54] (standardized validated instrument used to measure general health status). All questionnaires for which a third party does not own the copyright can be found in Multimedia Appendices 3 [37], 4 [38], 5 [39], 6 [40], 7 [41], 8 [45], 9 [50], 10 [51], and 11 [54].

### Retention and Withdrawal

Participants had the right to withdraw from the trial at any time. Participants were considered as withdrawn from the trial if they withdrew from the study intervention voluntarily or if they failed to return for follow-up assessments. Participants could also be removed by the principal investigator if this was considered necessary for medical reasons or due to ineligibility arising during the study (eg, pregnancy). Reasons for withdrawal were recorded, and their GP was notified with recommendations for follow-up care where appropriate. Participants who withdrew continued to have weight and HbA_1c_ collected from their routine diabetes clinic or GP visits for the duration of the 12-month trial unless they did not consent to this at baseline. No incentives were provided to the participants to promote retention and follow-up.

### Adverse Events

All adverse events in the 12-month study period were recorded by following the Good Clinical Practice and Health Research Authority processes. Nonserious adverse events such as constipation, fatigue, or hair loss were recorded when participants informed the trial team.

### Qualitative Evaluation

In-depth semistructured interviews were conducted with a subset of 10 ILED and 10 CLED participants at the end of the active weight loss phase (ILED week 28, CLED week 12). HCPs (n=6) delivering the programs were also interviewed near study completion. All interviews were conducted by an independent research assistant trained in qualitative interview techniques. Interviews explored the participant and HCP experiences of the ILED and CLED programs and the use of the Oviva app. Purposive sampling was used to select trial participants with a range of success in terms of actual weight loss and HbA_1c_ reduction, as well as participants' perceived success. Participants with ethnicities other than White-British were included where possible, with a fairly even split between men and women. All participants gave written informed consent to be interviewed and were assured that their data would be anonymized. The interviews were audiorecorded and transcribed verbatim for thematic analysis.

### Sample Size and Statistical Analysis

The total number of participants recruited for the study was 79. The sample size was selected to allow an estimate of uptake within ±6.6% of the target uptake of 10% [55] with 95% confidence while allowing the research team to obtain sufficient data on the feasibility and potential efficacy of the ILED and CLED.

This study will not undertake significance tests of changes to the primary outcome measures. Descriptive, graphical (summary), and basic inferential statistics of outcomes will be presented as appropriate, for example, frequencies and percentages, mean and SD, or median and quartiles. Confidence intervals (95%) will be calculated to show the change from baseline in the outcomes for each group.

Questionnaires used as outcome measures are quantitative and will be analyzed using appropriate descriptive statistics as per standard.

Changes to diabetes medication will be presented using the medication effect score (MES). The MES for a participant is the sum of the MES for each of their individual medications, where MES=actual drug dose/maximum drug dose×drug mean adjustment factor. A decrease in MES corresponds to a decrease in the use of diabetes medications [56]. Changes to BP medications will be presented using a Treatment Intensity Score (TIS) defined as the actual drug dose/maximum drug dose [57]. The TIS for a participant is the sum of the TIS for each of their medications, and a decrease in TIS indicates a decrease in BP medications.

An ITT analysis using multiple imputations will conducted for percentage weight loss, HbA_1c_, and MES. All other outcomes will be presented only for those who completed the trial.

### Data Management

Data were recorded on hard copy case report forms and subsequently transferred to a database with ranges and programmed validation checks to aid reliable data entry. Data are held on secure servers at MFT.

### Trial Steering Committee and Trial Management Group

The trial steering committee provided oversight for participant safety and included 2 co-principal investigators (BI and MH), an external endocrinologist and independent external advisor with experience of LEDs in the management of T2D. The committee met every 3 months to review and ensure the safety aspects of the trial. The trial management group, which comprised the chief investigators, diabetes specialist dietitians, a diabetes specialist nurse, and research nurses, evaluated all adverse events. The trial could have been stopped by the sponsor, chief investigators, or the trial management group or trial steering committee on the basis of new safety information or for other reasons given by the research ethics committee, but this was not required. The trial was subject to inspection and audit by MFT as the trial sponsor.

## Results

The project was funded in May 2017, ethical approval was obtained in August 2017, and enrollment began in February 2018. In total, 79 participants were recruited and randomized to the ILED (n=39) and CLED (n=40) arms of the trial. Data collection was completed in February 2020. Data analysis began in June 2020, and the first results are expected to be submitted for publication in 2021.

## Discussion

This is the first study to compare an ILED with an isocaloric CLED to achieve and maintain weight loss and normoglycemia among patients with T2D and overweight and obesity. The study will inform the acceptability and potential efficacy of high-frequency remote follow-up in patients with T2D and overweight and obesity undertaking low energy diets. It will also contribute to the limited data on the safety and efficacy of patients with T2D on insulin undertaking a ILED or CLED. The study did not have a standard or best practice control group for comparison and was not powered to show statistical differences between the groups. However, the planned quantitative and qualitative analyses will assess the feasibility of the programs and inform the case for a future definitive trial.

## Appendix

Multimedia Appendix 1 Monitoring and medication management plan.

Multimedia Appendix 2 Support provided by the multidisciplinary team.

Multimedia Appendix 3 Binge Eating Scale.

Multimedia Appendix 4 Weight efficacy lifestyle questionnaire short-form.

Multimedia Appendix 5 Generalized anxiety disorder scale.

Multimedia Appendix 6 Patient health questionnaire.

Multimedia Appendix 7 Alcohol use disorders identification test.

Multimedia Appendix 8 Physical activity readiness questionnaire.

Multimedia Appendix 9 Mediterranean diet score.

Multimedia Appendix 10 Scottish physical activity questionnaire.

Multimedia Appendix 11 Eq-5d-3l.

## Abbreviations

ADDQoL: Audit of Diabetes-Dependent Quality of Life
AUDIT: Alcohol Use Disorders Identification Test
BES: Binge Eating Scale
BP: blood pressure
CLED: continuous low-energy diet
DiRECT: Diabetes Remission Clinical Trial
GAD: Generalized Anxiety Disorder
GP: general practitioner
HbA_1c_: glycated hemoglobin
HCP: health care professional
ILED: intermittent low-energy diet
IMD: Index of Multiple Deprivation
ITT: intention-to-treat
LED: low-energy diet
MES: medication effect score
MFT: Manchester University NHS Foundation Trust
MIDDAS: Manchester Intermittent versus Daily diet Diabetes App Study
NHS: National Health Service
PA: physical activity
PAR-Q: Physical Activity Readiness Questionnaire
PHQ-9: Patient Health Questionnaire-9
RCT: randomized controlled trial
T2D: type 2 diabetes
TIS: Treatment Intensity Score
WEL-SF: Weight Efficacy Lifestyle Questionnaire Short Form
