# Supplementary material for: Intermittent Versus Continuous Low-Energy Diet in Patients With Type 2 Diabetes: Protocol for a Pilot Randomized Controlled Trial
Source: JMIR Res Protoc. 2021 Mar 19;10(3):e21116. doi: 10.2196/21116 (PMC8088860; doi:10.2196/21116)
Supplement: Multimedia Appendix 1 [file resprot_v10i3e21116_app1.docx]

## Schedule for glucose monitoring

**Table S1: Schedule for glucose monitoring**

| **Dietary program** | **Continuous low energy diet (CLED)** | **Intermittent low energy diet (ILED)** |
| --- | --- | --- |
| **Active weight loss phase** | - **Insulin treated patients:**   7 x daily (before and 2 hours after each meal and at bedtime)   - **Sulphonylurea (if introduced during study) treated patients:**   4 x daily (before each meal and at bedtime)   - **Non hypoglycaemia inducing agents:**   1 x daily (fasting) | - **Insulin treated patients:**   7 x daily (before and 2 hours after each meal and at bedtime) during 2 low energy days and 4 x daily (before each meal and at bedtime) during 5 days of the mediterranean diet.   - **Sulphonylurea (if introduced during study) treated patients:**   4 x daily (before each meal and at  bedtime)   - **Non hypoglycaemia inducing agents:**   1 x daily (fasting) |
| **Diet reintroduction phase** | - **Insulin treated patients:**   7 x daily (before and 2 hours after each meal and at bedtime**)**   - **Sulphonylurea (if introduced**   **during study) treated patients:**  4 x daily (before each meal and  at bedtime**)**   - **Non hypoglycaemia inducing agents:**   1 x daily (fasting) | N/A |
| **Weight maintenance**  **/continued weight loss phase** | - **Insulin treated patients**:   4 x daily (before each meal and  at bedtime)   - **Sulphonylurea (if introduced**   **during study) treated patients**:  2 x daily (before morning and evening meal)   - **Non hypoglycaemia inducing agents:**   1 x daily (fasting) | - **Insulin treated patients:**   7 x daily (before and 2 hours after each meal and at bedtime during 1 or 2 low energy days and 4 x daily (before each meal and at bedtime) during 5 or 6 days of the mediterranean diet.   - **Sulphonylurea (if introduced during**   **study) treated patients:**  2 x daily (before morning and evening  meal)   - **Non hypoglycaemia inducing agents:**   1 x daily (fasting) |
| **Relapse** | As in active weight loss phase | As in active weight loss phase |

## Schedule for blood pressure monitoring

Participants who were hypertensive or on antihypertensive medications at baseline were asked to measure their blood pressure once a month at their general practice or at home and to report their reading(s) to the trial team through the Oviva app or via email / telephone.

## Medication management plan

The following medication management plan applied from day one of the ILED / CLED programs and during relapse management. The ILED regimen was adapted from a protocol tested in a recent ILED trial. The CLED regimen was devised by the research team.[1]

**Table S2: Medication management plan**

|  | **CLED** | **ILED** |
| --- | --- | --- |
| **Non-insulin diabetes medications** | - Stop all diabetes medications on day 1, except for metformin. | - If HbA1c <53mmol/mol stop all diabetes medications likely to cause hypoglycaemia on day 1 for the duration of the trial. Metformin, DPP-4 inhibitors, GLP-1 analogues and SGLT2 inhibitors to remain unchanged. - If HbA1c ≥53mmol/mol and <86mmol/mol stop all diabetes medications likely to cause hypoglycaemia on the two low energy days each week. Metformin, gliptins, GLP-1 analogues and SGLT2 inhibitors to remain unchanged. - If HbA1c ≥86mmol/mol all diabetes medications remain unchanged. |
| **Insulin** | - Stop insulin if baseline HbA1c <86 mmol/mol - Reduce background and rapid acting insulin by 50% if HbA1c ≥86 mmol/mol, continue oral hypoglycemic agents and metformin.   Above regimen to be continued during the weight management / continued weight loss phase unless blood glucose readings indicate that changes are needed. | - If HbA1c <53 mmol/mol stop background and rapid acting insulin on day 1 for the duration of the active weight loss phase. - If HbA1c ≥53mmol/mol and <86mmol/mol stop background and rapid acting insulin on the 2 low energy days each week only. - If HbA1c ≥86mmol/mol insulin reduced by 50% on the two low energy days each week. |
| **Management of hypo /hyper-glycaemia for participants on insulin** | - Basal o.d insulin: for any hypoglycaemia episode (blood glucose <4mmol/l) reduce insulin by 10%-20%. - Basal b.d. insulin: in the event of daytime hypoglycaemia reduce morning insulin by 10%-20% and in the event of nighttime hypoglycaemia reduce evening insulin by 10%-20%. - Pre-mixed insulins: for nighttime hypoglycaemia, reduce pre-dinner insulin by 10-20% and for two or more day time hypoglycaemia episodes reduce morning insulin dose by 10%-20%. - Basal/bolus: for nighttime hypoglycaemia, reduce pre-bed basal insulin by 10-20% and for two or more pre-meal hypos, reduced morning basal or corresponding rapid acting insulin by 10-20%. - The same rules apply in reverse if the participant's blood glucose is running above target, with increasing insulin doses. | |
| **Hypertensive medications** | - All antihypertensive medication to be stopped. - For patients with systolic BP >165 mm Hg to consider continuing one or more antihypertensive medication. | - Diuretics to be stopped. - Other medications for hypertension to be continued and tapered according to blood pressure values. |

## References

1. Carter S, Clifton PM, Keogh JB. Intermittent energy restriction in type 2 diabetes: A short discussion of medication management. World J Diabetes 2016;7(20):627. PMID: 28031781

This is a Multimedia Appendix to a full manuscript published in the JMIR Research Protocols journal.
For full copyright and citation information see http://dx.doi.org/10.2196/jmir.21116
