# Supplementary material for: Intermittent Versus Continuous Low-Energy Diet in Patients With Type 2 Diabetes: Protocol for a Pilot Randomized Controlled Trial
Source: JMIR Res Protoc. 2021 Mar 19;10(3):e21116. doi: 10.2196/21116 (PMC8088860; doi:10.2196/21116)
Supplement: Multimedia Appendix 2 [file resprot_v10i3e21116_app2.docx]

|  | **Week 1-28** | | | | | | | | | | | | | | | | | | | | | | | | | | | | |
| --- | --- | --- | --- | --- | --- | --- | --- | --- | --- | --- | --- | --- | --- | --- | --- | --- | --- | --- | --- | --- | --- | --- | --- | --- | --- | --- | --- | --- | --- |
|  | Baseline | 1 | 2 | 3 | 4 | 5 | 6 | 7 | 8 | 9 | 10 | 11 | 12 | 13 | 14 | 15 | 16 | 17 | 18 | 19 | 20 | 21 | 22 | 23 | 24 | 25 | 26 | 27 | 28 |
| ILED |  | Active weight loss phase | | | | | | | | | | | | | | | | | | | | | | | | | | | |
| CLED |  | Active weight loss phase | | | | | | | | | | | | Weight maintenance/continued weight loss phase | | | | | | | | | | | | | | | |
| Dietitian^a^ | F/T | A | A | A | A | A | A | A | T | A | A | A | T |  | A |  | A |  | A |  | A |  | A |  | A |  | A |  | T |
| Nurse^b^ | T | T  /  A |  |  | T  /  A |  |  |  | T  /  A |  | T  /  A |  | T  /  A | T / A support as clinically needed | | | | | | | | | | | | | | | |
| Exercise Specialist | T |  |  |  |  |  |  |  | T |  |  |  | T |  |  |  |  |  |  |  |  |  |  |  |  |  |  |  | T |
| Psychologist^c^ | F |  |  |  |  |  |  | F/T/A |  | F / T / A once in weeks 9-12 | | | | ‘A’ support as needed throughout  + F / T support offered during  relapse (CLED only in weeks 1-28) | | | | | | | | | | | | | | | |

## Support provided by the multidisciplinary team Table S1: support provided by the multidisciplinary team in weeks 1-28

## Table S2: support provided by the multidisciplinary team in weeks 29-52

|  | **Weeks 29-52** | | | | | | | | | | | | | | | | | | | | | | | |
| --- | --- | --- | --- | --- | --- | --- | --- | --- | --- | --- | --- | --- | --- | --- | --- | --- | --- | --- | --- | --- | --- | --- | --- | --- |
|  | 29 | 30 | 31 | 32 | 33 | 34 | 35 | 36 | 37 | 38 | 39 | 40 | 41 | 42 | 43 | 44 | 45 | 46 | 47 | 48 | 49 | 50 | 51 | 52 |
| ILED | Weight maintenance/continued weight loss phase | | | | | | | | | | | | | | | | | | | | | | | |
| CLED | Weight maintenance/continued weight loss phase | | | | | | | | | | | | | | | | | | | | | | | |
| Dietitian^a^ |  |  |  | A |  |  |  | A |  |  |  | A |  |  |  | A |  |  |  | A |  |  |  | A |
| Nurse^b^ | T / A support as clinically needed | | | | | | | | | | | | | | | | | | | | | | | |
| Exercise Specialist |  |  |  |  |  |  |  |  |  |  |  |  |  |  |  |  |  |  |  |  |  |  |  | T |
| Psychologist^c^ | ‘A’ support as needed throughout  + F / T support offered during relapse | | | | | | | | | | | | | | | | | | | | | | | |

^a.^ Additional dietetic support during relapse
^b.^ The diabetes specialist nurse provided support for patients who were on diabetes medications other than metformin, on anti-hypertensives or who were hypertensive at baseline.
^c.^ Psychologist support if eligible / needed
Abbrevations: ILED = Intermittent Low Energy Diet program, CLED = Continuous Low Energy Diet Program, F = face-to-face support, T = Telephone support, A = App support
