# Supplementary material for: Intermittent Versus Continuous Low-Energy Diet in Patients With Type 2 Diabetes: Protocol for a Pilot Randomized Controlled Trial
Source: JMIR Res Protoc. 2021 Mar 19;10(3):e21116. doi: 10.2196/21116 (PMC8088860; doi:10.2196/21116)
Supplement: Multimedia Appendix 3 [file resprot_v10i3e21116_app3.docx]

Baseline / 6M / 12M

Date: ……/……/…...

Checked: ⬜ Initial…………………….

Participant Initials:……………….
Study Number: ……………….
RM2 Number:………………………….

**Questionnaire: BES**

**Instructions Below are groups of statements. Read all of the statements in each group and circle which option best describes the way you feel about your eating behaviour.**

**Number 1**

1. I don’t feel self-conscious about my weight or body size when I’m with others.
2. I feel concerned about how I look to others, but it normally does not make me feel disappointed with myself.
3. I do get self-conscious about my appearance and weight which makes me feel disappointed in myself.
4. I feel very self-conscious about my weight and frequently, I feel intense shame and disgust for myself. I try to avoid social contacts because of my self-consciousness.

**Number 2**

1. I don’t have any difficulty eating slowly in the proper manner.
2. Although I seem to “gobble down” foods, I don’t end up feeling stuffed because of eating too much.
3. At times, I tend to eat quickly and then, I feel uncomfortably full afterwards.
4. I have the habit of bolting down my food, without really chewing it. When this happens I usually feel uncomfortably stuffed because I’ve eaten too much.

**Number 3**

1. I feel capable to control my eating urges when I want to.
2. I feel like I have failed to control my eating more than the average person.
3. I feel utterly helpless when it comes to feeling in control of my eating urges.
4. Because I feel so helpless about controlling my eating I have become very desperate about trying to get in control.

**Number 4**

1. I don't have the habit of eating when I'm bored.
2. I sometimes eat when I'm bored, but often I'm able to "get busy" and get my mind off food.
3. I have a regular habit of eating when I'm bored, but occasionally, I can use some other activity to get my mind off eating.
4. I have a strong habit of eating when I'm bored. Nothing seems to help me break the habit.

**Number 5**

1. I'm usually physically hungry when I eat something.
2. Occasionally, I eat something on impulse even though I really am not hungry.
3. I have the regular habit of eating foods that I might not really enjoy, to satisfy a hungry feeling even though physically, I don't need the food.

This is a Multimedia Appendix to a full manuscript published in the JMIR Research Protocols journal.

For full copyright and citation information see http://dx.doi.org/10.2196/jmir.21116

1. Even though I'm not physically hungry, I get a hungry feeling in my mouth that only seems to be satisfied when I eat a food, like a sandwich, that fills my mouth. Sometimes, when I eat the food to satisfy my mouth hunger, I then spit the food out so I won't gain weight.

**Number 6**

1. I don’t feel any guilt or self-hate after I overeat.
2. After I overeat, occasionally I feel guilt or self-hate.
3. Almost all the time I experience strong guilt or self-hate after I overeat.

**Number 7**

1. I don’t lose total control of my eating when dieting even after periods when I overeat.
2. Sometimes when I eat a “forbidden food” on a diet, I feel like I “blew it” and eat even more.
3. Frequently, I have the habit of saying to myself, “I’ve blown it now, why not go all the way” when I overeat on a diet. When that happens I eat even more.
4. I have a regular habit of starting strict diets for myself, but I break the diets by going on an eating binge. My life seems to be either a “feast” or “famine.”

**Number 8**

1. I rarely eat so much food that I feel uncomfortably stuffed afterwards.
2. Usually about once a month, I eat such a quantity of food, I end up feeling very stuffed.
3. I have regular periods during the month when I eat large amounts of food, either at mealtime or at snacks.
4. I eat so much food that I regularly feel quite uncomfortable after eating and sometimes a bit nauseous.

**Number 9**

1. My level of calorie intake does not go up very high or go down very low on a regular basis.
2. Sometimes after I overeat, I will try to reduce my caloric intake to almost nothing to compensate for the excess calories I’ve eaten.
3. I have a regular habit of overeating during the night. It seems that my routine is not to be hungry in the morning but overeat in the evening.
4. In my adult years, I have had week-long periods where I practically starve myself. This follows periods when I overeat. It seems I live a life of either “feast or famine.”

**Number 10**

1. I usually am able to stop eating when I want to. I know when “enough is enough.”
2. Every so often, I experience a compulsion to eat which I can’t seem to control.
3. Frequently, I experience strong urges to eat which I seem unable to control, but at other times I can control my eating urges.
4. I feel incapable of controlling urges to eat. I have a fear of not being able to stop eating voluntarily.

**Number 11**

1. I don’t have any problem stopping eating when I feel full.
2. I usually can stop eating when I feel full but occasionally overeat leaving me feeling uncomfortably stuffed.
3. I have a problem stopping eating once I start and usually I feel uncomfortable stuffed after I eat a meal.
4. Because I have a problem not being able to stop eating when I want, I sometimes have to induce vomiting to relieve my stuffed feeling.

**Number 12**

1. I seem to eat just as much when I’m with others (family, social gatherings) as when I’m by myself.
2. Sometimes, when I’m with other persons, I don’t eat as much as I want to eat because I’m self-conscious about my eating.
3. Frequently, I eat only a small amount of food when others are present, because I’m very embarrassed about my eating.
4. I feel so ashamed about overeating that I pick times to overeat when I know no one will see me. I feel like a “closet eater.”

**Number 13**

1. I eat three meals a day with only an occasional between meal snack.
2. I eat 3 meals a day, but I also normally snack between meals.
3. When I am snacking heavily, I get in the habit of skipping regular meals.
4. There are regular periods when I seem to be continually eating, with no planned meals.

**Number 14**

1. I don’t think much about trying to control unwanted eating urges.
2. At least some of the time, I feel my thoughts are pre-occupied with trying to control my eating urges.
3. I feel that frequently I spend much time thinking about how much I ate or about trying not to eat anymore.
4. It seems to me that most of my waking hours are pre-occupied by thoughts about eating or not eating. I feel like I’m constantly struggling not to eat.

**Number 15**

1. I don’t think about food a great deal.
2. I have strong cravings for food but they last only for brief periods of time.
3. I have days when I can’t seem to think about anything else but food.
4. Most of my days seem to be pre-occupied with thoughts about food. I feel like I live to eat.

**Number 16**

1. I usually know whether or not I’m physically hungry. I take the right portion of food to satisfy me.
2. Occasionally, I feel uncertain about knowing whether or not I’m physically hungry. At these times it’s hard to know how much food I should take to satisfy me.
3. Even though I might know how many calories I should eat, I don’t have any idea what is a “normal” amount of food for me.
