# Supplementary material for: Intermittent Versus Continuous Low-Energy Diet in Patients With Type 2 Diabetes: Protocol for a Pilot Randomized Controlled Trial
Source: JMIR Res Protoc. 2021 Mar 19;10(3):e21116. doi: 10.2196/21116 (PMC8088860; doi:10.2196/21116)
Supplement: Multimedia Appendix 4 [file resprot_v10i3e21116_app4.doc]

Baseline / 6M / 12M

Date: ……/……/…...

Checked: ⬜ Initial…………………….

Participant Initials: ……………….
Study Number: ……………….
RM2 Number: ………………………….

| **Weight Efficacy Lifestyle Questionnaire Short-Form (WEL-SF)**  **Read each situation below and decide how confident (or certain) you are that you will be able to resist overeating in each of the difficult situations. On a scale of 0 (not confident) to 10 (very confident), choose ONE number that reflects how confident you feel now about being able to successfully resist the desire to overeat. Write this number next to each item.** | | | | | | | | | | | |
| --- | --- | --- | --- | --- | --- | --- | --- | --- | --- | --- | --- |
| 0  **Not** at all confident | 1 | 2 | 3 | 4 | 5 | 6 | 7 | | 8 | 9 | 10  **Very** Confident |
| **I AM CONFIDENT THAT:** | | | | | | | | **Confidence Number** | | | |
| 1. I can resist overeating when I am anxious (or nervous). | | | | | | | | _________ | | | |
| 2. I can resist overeating on the weekend. | | | | | | | | _________ | | | |
| 3. I can resist overeating when I am tired. | | | | | | | | _________ | | | |
| 4. I can resist overeating when I am watching TV (or using the computer). | | | | | | | | _________ | | | |
| 5. I can resist overeating when I am depressed (or down). | | | | | | | | _________ | | | |
| 6. I can resist overeating when I am in a social setting (or at a party). | | | | | | | | _________ | | | |
| 7. I can resist overeating when I am angry (or irritable). | | | | | | | | _________ | | | |
| 8. I can resist overeating when others are pressuring me to eat. | | | | | | | | _________ | | | |

Ames GE, Heckman MG, Grothe KB, Clark MM (2012) Eating self-efficacy: Development of a short-form WEL. *Eating Behaviours* 13: 375-378

This is a Multimedia Appendix to a full manuscript published in the JMIR Research Protocols journal.

For full copyright and citation information see http://dx.doi.org/10.2196/jmir.21116
