# Supplementary material for: Intermittent Versus Continuous Low-Energy Diet in Patients With Type 2 Diabetes: Protocol for a Pilot Randomized Controlled Trial
Source: JMIR Res Protoc. 2021 Mar 19;10(3):e21116. doi: 10.2196/21116 (PMC8088860; doi:10.2196/21116)
Supplement: Multimedia Appendix 6 [file resprot_v10i3e21116_app6.docx]

Participant Initials: ……………….
Study Number: ……………….
RM2 Number: ………………………….

Baseline / 2M / 3M / 6M / 12M

Date: ……/……/…...

Checked: ⬜ Initial…………………….

This is a Multimedia Appendix to a full manuscript published in the JMIR Research Protocols journal.

For full copyright and citation information see http://dx.doi.org/10.2196/jmir.21116

**Questionnaire: PHQ-9**

**(Please circle your answers)**

**
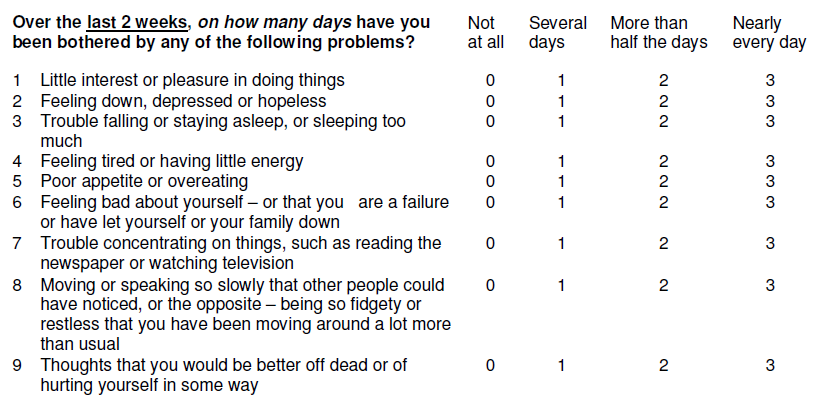
**

Spitzer RL, Kroenke K, Williams JB, Lowe B (2006) A brief measure for assessing Generalised Anxiety Disorder: the GAD-7. Arch Intern Med 166(10): 1092-1097


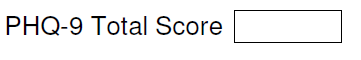


Kroenke K, Spitzer RL, Williams JB (2001) The PHQ-9: validity of a brief depression severity measure. *J Gen Intern Med 16(9): 06- 613.*
