# Supplementary material for: Intermittent Versus Continuous Low-Energy Diet in Patients With Type 2 Diabetes: Protocol for a Pilot Randomized Controlled Trial
Source: JMIR Res Protoc. 2021 Mar 19;10(3):e21116. doi: 10.2196/21116 (PMC8088860; doi:10.2196/21116)
Supplement: Multimedia Appendix 7 [file resprot_v10i3e21116_app7.doc]

Baseline / 6M / 12M

Date: ……/……/…...

Checked: ⬜ Initial…………………….

Participant Initials: ……………….
Study Number: ……………….
RM2 Number: ………………………….

This is a Multimedia Appendix to a full manuscript published in the JMIR Research Protocols journal.

For full copyright and citation information see http://dx.doi.org/10.2196/jmir.21116

**AUDIT – C Questionnaire**

**This is one unit of alcohol…**

**…and each of these is more than one unit**

| **Questions** | **Scoring system** | | | | | **Your score** |
| --- | --- | --- | --- | --- | --- | --- |
|  | **0** | **1** | **2** | **3** | **4** |  |
| How often do you have a drink containing alcohol? | Never | Monthly  or less | 2 - 4 times per month | 2 - 3 times per week | 4+ times per week |  |
| How many units of alcohol do you drink on a typical day when you are drinking? | 1 -2 | 3 - 4 | 5 - 6 | 7 - 9 | 10+ |  |
| How often have you had 6 or more units if female, or 8 or more if male, on a single occasion in the last year? | Never | Less than monthly | Monthly | Weekly | Daily or almost daily |  |

**Scoring:**

**SCORE**

A total of 5+ indicates increasing or higher risk drinking.

An overall total score of 5 or above is AUDIT-C positive.

**Score from AUDIT- C (other side)**

**SCORE**

**Remaining AUDIT questions**

| **Questions** | **Scoring system** | | | | | **Your score** |
| --- | --- | --- | --- | --- | --- | --- |
|  | **0** | **1** | **2** | **3** | **4** |  |
| How often during the last year have you found that you were not able to stop drinking once you had started? | Never | Less than monthly | Monthly | Weekly | Daily or almost daily |  |
| How often during the last year have you failed to do what was normally expected from you because of your drinking? | Never | Less than monthly | Monthly | Weekly | Daily or almost daily |  |
| How often during the last year have you needed an alcoholic drink in the morning to get yourself going after a heavy drinking session? | Never | Less than monthly | Monthly | Weekly | Daily or almost daily |  |
| How often during the last year have you had a feeling of guilt or remorse after drinking? | Never | Less than monthly | Monthly | Weekly | Daily or almost daily |  |
| How often during the last year have you been unable to remember what happened the night before because you had been drinking? | Never | Less than monthly | Monthly | Weekly | Daily or almost daily |  |
| Have you or somebody else been injured as a result of your drinking? | No |  | Yes, but not in the last year |  | Yes, during the last year |  |
| Has a relative or friend, doctor or other health worker been concerned about your drinking or suggested that you cut down? | No |  | Yes, but not in the last year |  | Yes, during the last year |  |

**Scoring:** 0 – 7 Lower risk, 8 – 15 Increasing risk,

**TOTAL = =**

16 – 19 Higher risk, 20+ Possible dependence

TOTAL Score equals

AUDIT C Score (above) +

Score of remaining questions
