# Supplementary material for: Intermittent Versus Continuous Low-Energy Diet in Patients With Type 2 Diabetes: Protocol for a Pilot Randomized Controlled Trial
Source: JMIR Res Protoc. 2021 Mar 19;10(3):e21116. doi: 10.2196/21116 (PMC8088860; doi:10.2196/21116)
Supplement: Multimedia Appendix 8 [file resprot_v10i3e21116_app8.docx]

Participant Initials: ……………….
Study Number: ……………….
RM2 Number: ………………………….

Baseline / 3M / 6M / 12M

Date: ……/……/…...

Checked: ⬜ Initial…………………….

This is a Multimedia Appendix to a full manuscript published in the JMIR Research Protocols journal.

For full copyright and citation information see http://dx.doi.org/10.2196/jmir.21116

**Physical Activity Readiness Questionnaire (PAR-Q)**

MIDDAS involves a home based, unsupervised exercise plan. Being more active is very safe for most people. However, some people may need to check with their doctor before starting the exercise plan. In order to help us assess whether it is safe for you to do the exercise plan, please read the following questions carefully and answer them to the best of your knowledge: circle **YES** or **NO**.

**If you answered “yes” to Q7, please briefly explain:**

| **1** | Has your doctor ever said that you have a heart condition and that you should only do physical activity recommended by a doctor? | **YES / NO** |
| --- | --- | --- |
| **2** | Do you feel pain in your chest when you do physical activity? | **YES / NO** |
| **3** | In the past month, have you had chest pain when you were not doing physical activity? | **YES / NO** |
| **4** | Do you lose your balance because of dizziness or do you ever lose consciousness? | **YES / NO** |
| **5** | Do you have a bone or joint problem (for example, back, knee or hip) that could be made worse by a change in your physical activity? | **YES / NO** |
| **6** | Is your doctor currently prescribing drugs (for example, water pills) for your blood pressure or heart condition? | **YES / NO** |
| **7** | Do you know of any other reason why you should not do physical activity? | **YES / NO** |

**Please print your name, sign and date below:**

**Participant name (print)**: ……………………………......................................

**Participant signature**: ……………………………......................................

**Date (DD/MM/YY):** ……………………………......................................

PLEASE NOTE: If your health changes so that you then answer YES to any of the above questions,
then please tell your exercise specialist and ask whether you should change your physical activity plan.

**Physical Activity Readiness Questionnaire (PAR-Q)**

**Witness name (Research Nurse) (print)**: …………………………….........................................

**Witness job title (Research Nurse) (print)**: ……………………………........................................

**Witness (Research Nurse) signature**: ……………………………........................................

**Date (DD/MM/YY):** ……………………………........................................

**Exercise specialist name (print)**: ………………………................................

**Exercise specialist signature**: ……………………………..........................

**Date (DD/MM/YY):** ……………………………......................................

**What to do next:**

- If you answered “**NO**” to ALL of the questions then you can undertake unrestricted physical activity starting off slowly and building up gradually.
- If you answered “**YES**” to one or more questions, then a member of the study team will provide you with a letter to take to your GP. Your GP needs to sign the letter to say what degree of exercise is safe for you to undertake, and you will need to return the letter to the MIDDAS study team before you can receive exercise advice. You can return the letter to the address below or ask your GP to fax the letter to **0161 291 4421**.

MIDDAS team

Research Dietitians

Nightingale Centre

Wythenshawe Hospital

Manchester

M23 9LT

Physical Activity Readiness Questionnaire (PAR-Q) & You (2002). Canadian Society for Exercise Physiology. <http://www.csep.ca/CMFiles/publications/parq/par-q.pdf>
