# Supplementary material for: Intermittent Versus Continuous Low-Energy Diet in Patients With Type 2 Diabetes: Protocol for a Pilot Randomized Controlled Trial
Source: JMIR Res Protoc. 2021 Mar 19;10(3):e21116. doi: 10.2196/21116 (PMC8088860; doi:10.2196/21116)
Supplement: Multimedia Appendix 9 [file resprot_v10i3e21116_app9.docx]

Participant Initials: ……………….
Study Number: ……………….

Baseline / 3M / 6M / 12M

Date: ……/……/…...

Checked: ⬜ Initial…………………….

**Healthy (Mediterranean) 12-point Diet Score**

| 1 | What oil or fat do you usually use for cooking? |  |
| --- | --- | --- |
| 2 | Are you more likely to choose wholegrain foods (breakfast cereals, rice and pasta) and wholegrain bread instead of low fibre cereals or white bread? | Yes / No  *(circle)* |
| 3 | How many servings of vegetables do you usually eat per day?  *(1 serving = 80g/3oz e.g. a bowl of salad, 3 florets of broccoli or cauliflower, one medium carrot)* | *(specify number)* |
| 4 | How many servings of fruit (including a maximum of one 150ml glass of pure fruit juice) do you usually consume per day?  *(1 serving = 80g/3oz e.g. 2 small fruits such as kiwis or satsumas, one medium fruit such as an apple or banana, or one large slice of a big fruit such as melon)* | *(specify number)* |
| 5 | How many servings of red meat i.e. pork, lamb, beef or meat products (ham, sausages, burgers etc.) do you usually eat per week?  *(1 serving = 100-150g/3.5-5oz e.g. 2 sausages, one quarter of a 500g pack of mince)* | *(specify number)* |
| 6 | How many servings of butter or cream do you usually have per day? *(1 serving =2 tsp)* | *(specify number)* |
| 7 | How many sugar sweetened beverages (e.g. full sugar fizzy drinks, full sugar squash, tea/coffee with sugar) do you drink per day? | *(specify number)* |
| 8 | How many servings of legumes (peas, beans, lentils and hummus) do you eat per week?  *(1 serving = 2 heaped tbsp. or 1 tbsp. of hummus)* | *(specify number)* |
| 9 | How many servings of fish or shellfish do you eat per week?  *(1 serving: 100-150g/3.5-5oz fish fillet, or 4-5 small fish e.g. sardines, or 200g shellfish)* | *(specify number)* |
| 10 | How many times (occasions) per week do you consume sweets, chocolate, pastries cakes, pies, cookies, scones, croissants, doughnuts or sweet desserts? | *(specify number)* |
| 11 | How many servings of nuts do you eat per week?  *(1 serving = 30 g)* | *(specify number)* |
| 12 | Are you more likely to chose chicken, turkey or meat alternatives (e.g. Quorn, tofu, soya) than red meat e.g. beef, pork, lamb? | Yes / No  *(circle)* |

Adapted from: Martinez-Gonzalez MA, Fernandez-Jarne E, Serrano-Martinez M, Wright M, Gomez-Gracia E (2004) Development of a short dietary intake questionnaire for the quantitative estimation of adherence to a cardioprotective Mediterranean diet. *Eur J Clin Nutr* 58 (11): 1550-1552

This is a Multimedia Appendix to a full manuscript published in the JMIR Research Protocols journal.

For full copyright and citation information see http://dx.doi.org/10.2196/jmir.21116
