# Supplementary material for: Intermittent Versus Continuous Low-Energy Diet in Patients With Type 2 Diabetes: Protocol for a Pilot Randomized Controlled Trial
Source: JMIR Res Protoc. 2021 Mar 19;10(3):e21116. doi: 10.2196/21116 (PMC8088860; doi:10.2196/21116)
Supplement: Multimedia Appendix 10 [file resprot_v10i3e21116_app10.doc]

Baseline / 3M / 6M / 12M

Date: ……/……/…...

Checked: ⬜ Initial…………………….

Participant Initials: ……………….
Study Number: ……………….
RM2 Number: ………………………….

# Scottish Physical Activity Questionnaire

• The following questions relate to your physical activity over the past week.

•  Please complete the diary for the past seven consecutive days.

# How to fill in your physical activity diary

•  Mark in the appropriate box the number of minutes spent doing a particular activity.

•  Only include activity of either **moderate** or **vigorous** intensity. Examples of what should and should not be included:

| **🗶** | **LIGHT INTENSITY** |
| --- | --- |
|  | Your heart rate and breathing rate are no different from what they are when you are standing, sitting etc. |
| **✓** | **MODERATE INTENSITY** |
|  | Your heart rate and breathing rate are faster than normal. You may also sweat a little. Brisk walking, sweeping or mopping are good examples of how you might feel. |
| **✓** | **VIGOROUS INTENSITY** |
|  | Your heart rate is much faster and you have to breathe deeper and faster than normal. You will probably sweat. Jogging or running are good examples of how you might feel. |

•  Please try and be as accurate as possible with your answers. For example you may have spent 2 hours shopping but only 1 hour of that time actually walking.

•  Be careful not to count the same activity twice. For example if you have been hill walking, only include this on the walking OR leisure activity section, not both.

**Look over your completed activity record:**

**If the week was not typical of how much activity you usually do, by how many minutes (more/less) and of which activity was it different?**

**More:** …………..**minutes of**………………………………................................

**Less:** ……...…...**minutes of**………………………………………………………

This is a Multimedia Appendix to a full manuscript published in the JMIR Research Protocols journal.

For full copyright and citation information see http://dx.doi.org/10.2196/jmir.21116

| **LEISURE TIME PHYSICAL ACTIVITY** | | |  |  |  |  |  |  |  |
| --- | --- | --- | --- | --- | --- | --- | --- | --- | --- |
| How many minutes did you spend each day: | | | Mon | Tue | Wed | Thu | Fri | Sat | Sun |
| **Walking outside of working hours?** | | |  |  |  |  |  |  |  |
| ***DO*** *include* | **✓** | walking to the shops, walking to work, walking the dog, stair-walking |  |  |  |  |  |  |  |
| ***DO NOT*** *include* | **🗶** | standing, sitting, driving, walking whilst **at** work, walking **round** the shops |  |  |  |  |  |  |  |
| **Manual labour outside of working hours?** | | |  |  |  |  |  |  |  |
| ***DO*** *include* | **✓** | cutting grass, decorating, washing car, DIY, digging |  |  |  |  |  |  |  |
|  |  |  |  |  |  |  |
| ***DO NOT*** *include* | **🗶** | weeding, planting, pruning |  |  |  |  |  |  |  |
| **Active housework?** | | |  |  |  |  |  |  |  |
| ***DO*** *include* | **✓** | vacuuming, scrubbing floors, bed making, hanging out washing |  |  |  |  |  |  |  |
|  |  |  |  |  |  |  |
| ***DO NOT*** *include* | **🗶** | sewing, dusting, washing dishes, preparing food |  |  |  |  |  |  |  |
| **Participating in a sport, leisure activity or training?** | | |  |  |  |  |  |  |  |
| ***DO*** *include* | **✓** | exercise classes, cycling, football, swimming, golf, jogging, running, athletics, dancing |  |  |  |  |  |  |  |
| ***DO NOT*** *include* | **🗶** | darts, snooker / pool, fishing, playing a musical instrument |  |  |  |  |  |  |  |
| **Other Physical Activity not listed above:** | | |  |  |  |  |  |  |  |
|  | | |  |  |  |  |  |  |  |
| **……………………………………………………………………………** | | |  |  |  |  |  |  |  |
| **MIDDAS Resistance Exercises ( No need to complete for the screening and assessment appointment)** | | | Mon | Tue | Wed | Thu | Fri | Sat | Sun |
| **How many minutes did you spend doing your resistance exercises each day?** | | |  |  |  |  |  |  |  |
|  |  |  |  |  |  |  |
| **PHYSICAL ACTIVITY AT WORK (■ Cross if you are not currently working)**  How many minutes did you spend each day: | | | Mon | Tue | Wed | Thu | Fri | Sat | Sun |
| **Walking whilst at work?** | | |  |  |  |  |  |  |  |
| ***DO*** *include* | **✓** | walking up or down stairs, to and from your desk, "doing the rounds" |  |  |  |  |  |  |  |
| ***DO NOT*** *include* | **🗶** | standing, sitting at desk etc; i.e. time spent not actually walking |  |  |  |  |  |  |  |
| **Manual labour whilst at work?** | | |  |  |  |  |  |  |  |
| ***DO*** *include* | **✓** | lifting, stacking shelves, climbing ladders, building work, cleaning |  |  |  |  |  |  |  |
|  |  |  |  |  |  |  |
| ***DO NOT*** *include* | **🗶** | sitting at desk, answering telephone, driving, check-out operation |  |  |  |  |  |  |  |

Lowther M, Mutrie N, Loughlan C, McFarlane C. Development of a Scottish physical activity questionnaire: a tool for use in physical activity interventions. Br J Sports Med 1999;33(4):244-9.
