# Supplementary material for: Intermittent Versus Continuous Low-Energy Diet in Patients With Type 2 Diabetes: Protocol for a Pilot Randomized Controlled Trial
Source: JMIR Res Protoc. 2021 Mar 19;10(3):e21116. doi: 10.2196/21116 (PMC8088860; doi:10.2196/21116)
Supplement: Multimedia Appendix 11 [file resprot_v10i3e21116_app11.docx]

Participant Initials: ……………….
Study Number: ……………….
RM2 Number: ………………………….

Baseline / 6M / 12M

Date: ……/……/…...

Checked: ⬜ Initial…………………….


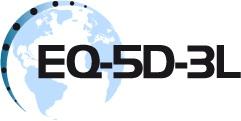


**Health Questionnaire**

**English version for the UK**

***(Validated for Ireland)***

This is a Multimedia Appendix to a full manuscript published in the JMIR Research Protocols journal.

For full copyright and citation information see http://dx.doi.org/10.2196/jmir.21116

*UK (English) © 1990 EuroQol Group EQ-5D™ is a trade mark of the EuroQol Group*

By placing a tick in one box in each group below, please indicate which statements best describe your own health state today.

**Mobility**

I have no problems in walking about

I have some problems in walking about

I am confined to bed

**Self-Care**

I have no problems with self-care

I have some problems washing or dressing myself

I am unable to wash or dress myself

**Usual Activities** *(e.g. work, study, housework, family or leisure activities)*

I have no problems with performing my usual activities

I have some problems with performing my usual activities I am unable to perform my usual activities

**Pain / Discomfort**

I have no pain or discomfort

I have moderate pain or discomfort

I have extreme pain or discomfort

**Anxiety / Depression**

I am not anxious or depressed

I am moderately anxious or depressed

I am extremely anxious or depressed

❑

❑

❑

❑

❑

❑

❑

❑

❑

❑

❑

❑

❑

❑

❑

2

*UK (English) © 1990 EuroQol Group EQ-5D™ is a trade mark of the EuroQol Group*

To help people say how good or bad a health state is, we have drawn a scale (rather like a thermometer) on which the best state you can imagine is marked 100 and the worst state you can imagine is marked 0.

We would like you to indicate on this scale how good or bad your own health is today, in your opinion. Please do this by drawing a line from the box below to whichever point on the scale indicates how good or bad your health state is today.


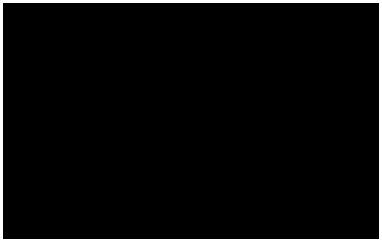


**Your own health**

**Your own health**

**state today**

3

Best imaginable health state

100


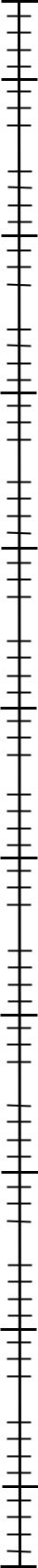


9
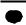
0

8
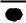
0

7
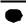
0

6
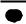
0

5
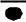
0

4
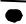
0

3
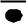
0

2
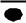
0

1
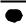
0

0

Worst imaginable health state

*UK (English) © 1990 EuroQol Group EQ-5D™ is a trade mark of the EuroQol Group*
